# National implementation of reperfusion for acute ischaemic stroke in England: How should services be configured? A modelling study

**DOI:** 10.1177/23969873211063323

**Published:** 2021-12-23

**Authors:** Michael Allen, Kerry Pearn, Gary A Ford, Phil White, Anthony G Rudd, Peter McMeekin, Ken Stein, Martin James

**Affiliations:** 1 3286 University of Exeter, Medical School and the National Institute for Health Research (NIHR) Applied Research Collaboration South West Peninsula (SWPenARC), Exeter, UK; 2 Radcliffe Department of Medicine, 568804 Oxford University and Oxford University Hospitals NHS Foundation Trust, Oxford, UK; 3 Translational and Clinical Research Institute, 12186 Newcastle University and Newcastle Upon Tyne Hospitals NHS Foundation Trust, Newcastle Upon Tyne, UK; 4 12196 Kings College London and Guy’s and St Thomas, NHS Foundation Trust, London, UK; 5 Faculty of Health and Life Sciences, 5995 Northumbria University, Newcastle Upon Tyne, UK; 6 159028 Royal Devon and Exeter NHS Foundation Trust, Exeter, UK

**Keywords:** Stroke, thrombolysis, thrombectomy, health services research

## Abstract

**Objectives:**

To guide policy when planning thrombolysis (IVT) and thrombectomy (MT) services for acute stroke in England, focussing on the choice between ‘mothership’ (direct conveyance to an MT centre) and ‘drip-and-ship’ (secondary transfer) provision and the impact of bypassing local acute stroke centres.

**Design:**

Outcome-based modelling study.

**Setting:**

107 acute stroke centres in England, 24 of which provide IVT and MT (IVT/MT centres) and 83 provide only IVT (IVT-only units).

**Participants:**

242,874 emergency admissions with acute stroke over 3 years (2015–2017).

**Intervention:**

Reperfusion delivered by drip-and-ship, mothership or ‘hybrid’ models; impact of additional travel time to directly access an IVT/MT centre by bypassing a more local IVT-only unit; effect of pre-hospital selection for large artery occlusion (LAO).

**Main outcome measures:**

Population benefit from reperfusion, time to IVT and MT, admission numbers to IVT-only units and IVT/MT centres.

**Results:**

Without pre-hospital selection for LAO, 94% of the population of England live in areas where the greatest clinical benefit, assuming unknown patient status, accrues from direct conveyance to an IVT/MT centre. However, this policy produces unsustainable admission numbers at these centres, with 78 out of 83 IVT-only units receiving fewer than 300 admissions per year (compared to 3 with drip-and-ship). Implementing a maximum permitted additional travel time to bypass an IVT-only unit, using a pre-hospital test for LAO, and selecting patients based on stroke onset time, all help to mitigate the destabilising effect but there is still some significant disruption to admission numbers, and improved selection of patients suitable for MT selectively reduces the number of patients who would receive IVT at IVT-only centres, challenging the sustainability of IVT expertise in IVT-only centres.

**Conclusions:**

Implementation of reperfusion for acute stroke based solely on achieving the maximum population benefit potentially leads to destabilisation of the emergency stroke care system. Careful planning is required to create a sustainable system, and modelling may be used to help planners maximise benefit from reperfusion while creating a sustainable emergency stroke care system.

## Introduction

In England, about 80,000 people are hospitalised each year with acute stroke,^1,2^ and over half of these people are left with long-term disability at great cost to individuals and society.^3^ Disability and institutionalisation after ischaemic stroke are significantly reduced by reperfusion treatments – intravenous thrombolysis (IVT)^4^ and mechanical thrombectomy (MT).^5^ IVT is a treatment that may be considered for all potentially disabling ischaemic strokes and in England and Wales about 40% of emergency stroke patients have known stroke onset and arrive within 4 h of onset.^6^ MT may be considered for all large artery occlusions (LAO), which are present in up to 40% of all acute ischaemic strokes.^7^

In 2019–2020, in the UK, these treatments were given to 11.7% and 1.8% of patients with acute stroke, respectively.^2^ The 2019 NHS England Long Term Plan^8^ set out the ambition that, by 2025, acute stroke units will deliver IVT to about 20% of patients, and MT to about 10%. To achieve such increases in reperfusion, the NHS England Long Term Plan acknowledges the need for the centralisation of hyperacute stroke care into fewer well-equipped and staffed hospitals, noting that metropolitan areas that have recently centralised stroke care have achieved improved outcomes.^9,10^

Two principal models for the provision of reperfusion are generally considered: the so-called *mothership*, where regional centres (sometimes referred to as comprehensive stroke centres) provide IVT and MT for all eligible suspected stroke patients, with ambulances bypassing local acute services; and *drip-and-ship*, where IVT is provided at smaller local units (sometimes referred to as acute stroke centres), followed by secondary transfer of selected patients to regional centres for MT. Currently, drip-and-ship is the predominant and commissioned model of provision in the UK. Reconfiguring hyperacute stroke services to a more centralised mothership model could increase direct access to MT for the eligible minority, but at the expense of increased travel times and delayed IVT for many patients; it could also substantially increase admissions to the regional MT centres, many of whom would be ineligible for reperfusion. Modelling and simulation has been suggested as being particularly useful in planning of stroke care for being able to generalise outside of local clinical studies.^11^ We therefore set out to examine the impact of the reperfusion ambitions in the NHS England Long Term Plan for stroke on population disability, and to model the optimal organisation of hyperacute stroke provision necessary to achieve these ambitions in a sustainable way.

## Method

### Location of thrombolysis and thrombectomy centres

The location of thrombolysis centres was taken as the 107 hyperacute stroke units in England providing thrombolysis in 2019.^2^ Within this number, the location of IVT/MT centres, in our base case model, was taken as the 24 neuroscience centres in England which are either providing or planning to provide MT^2^ (in a recent survey of stroke units across all the UK, 24 out of 28 planned MT centres were operational).^12^

### Admission numbers and travel times for patients

Patients are located to one of 32,833 Lower Super Output Areas (LSOA) in England. Hospital admissions for stroke in England per LSOA for 3 years 2015–2017 were obtained from NHS Hospital Episodes Statistics, including 242,874 patients coded with an emergency admission of confirmed ischaemic or haemorrhagic stroke (primary diagnosis ICD-10 I61, I63, I64). When predicting the number of MT procedures, we have usually assumed that the proportion of confirmed stroke patients eligible for MT in the UK is 10% of all stroke, as previously estimated,^13^ and we use the NHS target for 20% IVT.^8^ In England and Wales, 88% of hospitalised stroke patients have an ischaemic stroke.^14^ Current use of IVT is 11% of all confirmed stroke patients across all of England, but this ranges between hospitals from 4 to 22%.^14^ As MT is currently being established, there are no steady-state figures yet for the use of MT in England. We model based on 107 active IVT centres in 2019, and 24 planned MT centres.

Travel times were estimated from all LSOA to all hospitals using Microsoft MapPoint. In a ‘drip and ship’ model, where an inter-hospital transfer may be required for MT, we assumed a net delay in MT of 60 min + inter-hospital transfer time (obtained from MapPoint).

### Clinical outcome

Clinical outcome from reperfusion is quantified in terms of a disability-free outcome 3 months after stroke (a modified Rankin Scale score of 0–1/6 describes either no or only minor non-disabling symptoms).^15^ We have published the detailed method and code used to estimate outcomes.^16^ This method is a development of the method of Holodinsky et al,^17^ and incorporates the known decay of effectiveness of IVT^4^ and MT^18^ over time.

For those patients with the prospect of treatment we assume it takes 60 min from stroke onset to the ambulance leaving the scene. Time to IVT is therefore 60 min + ambulance travel time + door-to-needle time (D2N). D2N times for IVT in the model are set to a standardised 40 min across all hospitals. For patients who attend an IVT/MT centre as their first admitting hospital, time to MT is given by 60 min + ambulance travel time + door-to-puncture time (D2P), where D2P for MT in the model are standardised to 90 min across all hospitals.

If a patient first attends a unit which does not provide MT, the time to MT incurs a further net delay and added inter-hospital travel time. The net delay used may be shorter than the actual door-in to door-out time at the first admitting hospital, as some process steps have already been completed on arrival at the MT centre. Previously, in a large clinical trial it was shown that patients receiving MT after transfer were treated 110 min later than patients admitted directly, 35 min of which was attributable to inter-hospital travel time, suggesting a net delay of 75 min + travel time.^19^ In our modelling we have anticipated some improvement over these historical results and have assumed a 60 min net delay in addition to the inter-hospital transfer travel time.

### Pre-hospital selection

In recent years, several methods for ambulance paramedics to select patients with LAO as potential candidates for MT have been developed.^20^ We have used the performance of one of the most widely studied, the RACE pre-hospital clinical diagnostic for LAO,^21^ to assess the potential impact of selection of patients most likely to benefit from direct transport to an IVT/MT centre, bypassing any nearer IVT-only centre. We model the effects of the RACE test based on published results,^21^ in which 32% of all stroke were large vessel occlusions, and a RACE scale ≥5 (henceforth referred to as a positive RACE test) had sensitivity 0.85, specificity 0.68, positive predictive value 0.42, and negative predictive value 0.94 for detecting LAO.

### Varying number and location of thrombectomy centres

For selecting locations of IVT/MT centres (in addition to the current 24 neuroscience centres) we have used a genetic algorithm as previously described.^22^ Briefly, we used a bespoke genetic algorithm based on NSGA-II.^23^ Genetic algorithms maintain a population of solutions. These solutions go through a series of generations where new solutions are formed by hybridising two existing solutions (with occasional random mutation). In each generation, the best solutions are kept. For selection of best solutions, we used a pareto-based method whereby, when there are multiple objectives, generated solutions are eliminated if another solution is equally as good in all optimisation parameters and is better in at least one parameter. The selected configurations were based on a range of optimisation parameters which seek to minimise travel times and to control admission numbers. National guidelines recommend a minimum number of admissions to an acute stroke unit, providing IVT, of 600 patients per year,^24^ coupled to the recommendation that travel time to hyperacute stroke care should be ideally 30 min or less, and no more than 60 min.^25^ A further competing priority is the minimum number of MT procedures to be performed by any given unit to maintain institutional expertise. In order to maintain the procedural skills of interventional teams and yet have sufficient specialists to staff a rota, we have used a minimum threshold of 150 MT procedures per year for an IVT/MT centre.^26^

## Results

### Benefit of IVT and MT – Drip and ship

**Figure 1.**
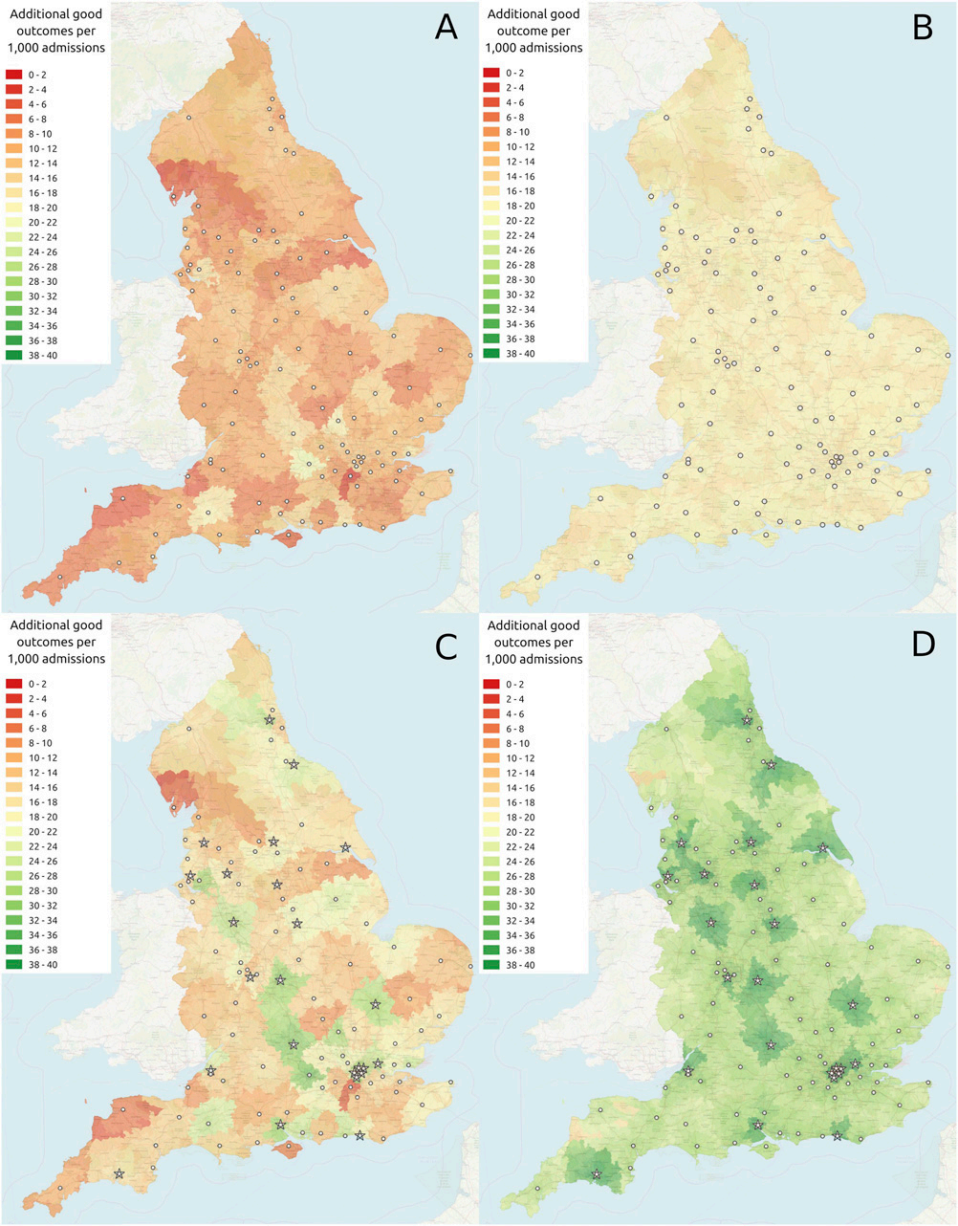
Geographical variation in clinical benefit (additional good outcomes / 1,000 admissions). a) IVT provided at 107 stroke units, with IVT rates for units taken from 2018 to 19 SSNAP report.^6^ b) IVT provided at 107 stroke units, with 20% IVT at all units. c) IVT provided at 107 stroke units, and MT also provided at the 24 neuroscience centres, with IVT rates for units taken from 2018 to 19 SSNAP report.^6^ d) IVT provided at 107 stroke units, and MT also provided at the 24 neuroscience centres, with 20% IVT at all units. Circles show IVT-only units, stars show MT centres, with 10% patients receiving MT.

We estimated net clinical benefit of a drip-and-ship model of care, where all patients are assumed to travel to their closest acute stroke unit (providing at least IVT) first, and travel onwards to their nearest IVT/MT centre if necessary. If current IVT rates (which average 11%, but range from 4 to 22%) are used, then the clinical benefit without and with 24/7 MT at 24 current neuroscience centres would be 10.9 and 18.0 additional disability-free outcomes/1000 admissions (we assume the rate of MT is half that of IVT). If uniform 20% IVT and 10% MT rates were achieved then clinical benefit without and with 24/7 MT at 24 neuroscience centres would be 18.9 and 31.1 additional disability-free outcomes/1000 admissions. Figure 1 shows the geographical variation in clinical benefit with these scenarios. First hospital admissions in this model range from 259 to 1964. If patients attend their closest unit first, 27% directly attend an IVT/MT centre first.

The rest of the analysis presented assumes uniform 20% IVT and 10% MT rates.

### Benefit of IVT and MT – Mothership

**Figure 2.**
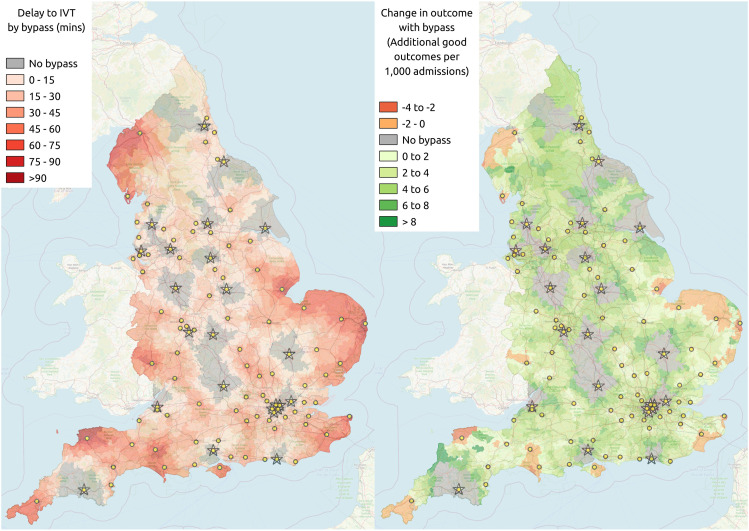
The effect of implementing a mothership model, where all patients bypass their more local IVT-only unit (circles) to directly attend one of the 24 MT centres (stars). Maps show delay in receiving IVT (left panel) and net change in the clinical benefit of reperfusion (right panel).

If all patients travelled to their nearest IVT/MT centres and bypassed a more local IVT-only unit, the delay in IVT and the effect on clinical outcome is shown in Figure 2. The net clinical benefit would be an additional 33.5 disability-free outcomes/1000 admissions. The mothership model therefore gives a modest net improvement in outcome over drip-and-ship; though importantly individual regions may have a poorer or better predicted outcome with a mothership model (ranging from a reduction of 4.0, to an increase of 13.8 additional disability-free outcomes/1000 admissions, with 5^th^ and 95^th^ percentiles of 0.0 and 6.0 increase). 66% of LSOA have an improvement of at least two additional disability-free outcomes/1000 admissions with a mothership model. First hospital admissions in this model range from 1228 to 6,183, with 22 out of 24 IVT/MT centres receiving more than 2000 patients per year.

### Destination based on most likely good outcome

**Figure 3.**
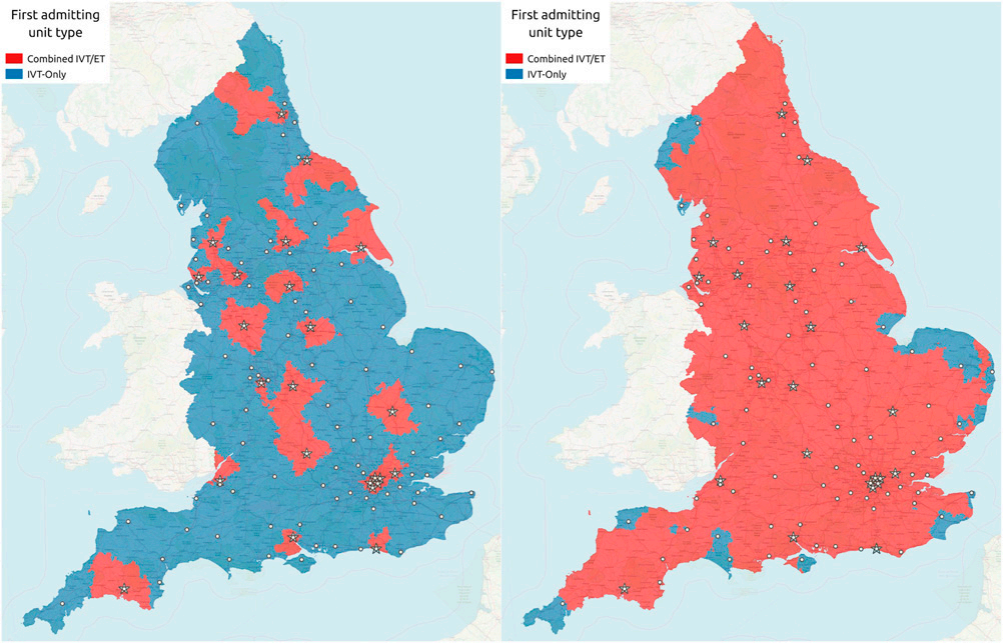
Catchment areas for either MT centres (stars) or IVT-only units (circles) if decision on conveyance to first stroke unit is made by either closest unit (left panel) or the destination that gives the highest probability of a good outcome (without any pre-hospital diagnostic; right panel). The model has 24 MT centres at the current neuroscience centres and 83 IVT-only units at the remaining current acute stroke centres.

Instead of all patients being conveyed to their closest acute stroke unit as in the drip-and-ship model, or all patients travelling directly to their nearest IVT/MT centre as in the mothership model, it is possible to make a patient-specific decision by using a prediction, in the absence of any diagnostic test for LAO, as to whether disability outcome is improved by bypassing a more local IVT-only unit and travelling further to directly attend an IVT/MT centre. In this model, net clinical benefit is predicted to increase to 33.6 additional disability-free outcomes/1000 admissions, a marginal improvement over an exclusive mothership model. Figure 3 shows the effect that this has on the catchment areas of IVT-only units and IVT/MT centres. Compared to the drip-and-ship model, the travel time to the first admitting stroke unit is, on average, increased from 19 to 34 min, but travel time to an IVT/MT centre is reduced from 94 to 42 min. Such a strategy causes significant disruption to admission numbers. Compared with the drip-and-ship model, the proportion of patients directly attending an IVT/MT centre rises from 27% to 94%, and average first admissions to an IVT/MT centre rises from 919 to 3187 (a 246% fold increase compared with all patients attending closest unit first), with the largest centre increasing from 1964 to 5578. Average first admissions to IVT-only units fall from 710 to 43 (a 94% reduction compared with all patients attending closest unit first), with 78 out of 83 IVT-only units receiving fewer than 300 admissions per year (compared to 3 with drip-and-ship).

This effect on admissions may be mitigated by only choosing patients for transport to a MT-capable centre only if they have a non-mild stroke (NIHSS > 5), have a known stroke onset time, and would expect to arrive at hospital within about 6 h of known stroke onset. National registry England and Wales^6^ show that 24.5% of emergency stroke admissions met these three criteria (these patients accounted for 73% of patients who received thrombolysis). If only these patients could be identified and selected for bypass of their local IVT unit, then the proportion of patients directly attending an IVT/MT centre would increase from 27% to 44%. Average first admissions to an IVT/MT centre would be 1474 (a 60% increase compared with all patients attending closest unit first), with the largest centre having 2725 admissions. Average first admissions to IVT-only units would be 549 (a 23% reduction compared with all patients attending closest unit first), with 6 IVT-only units receiving fewer than 300 admissions per year.

### Destination based on maximum additional travel time

**Figure 4.**
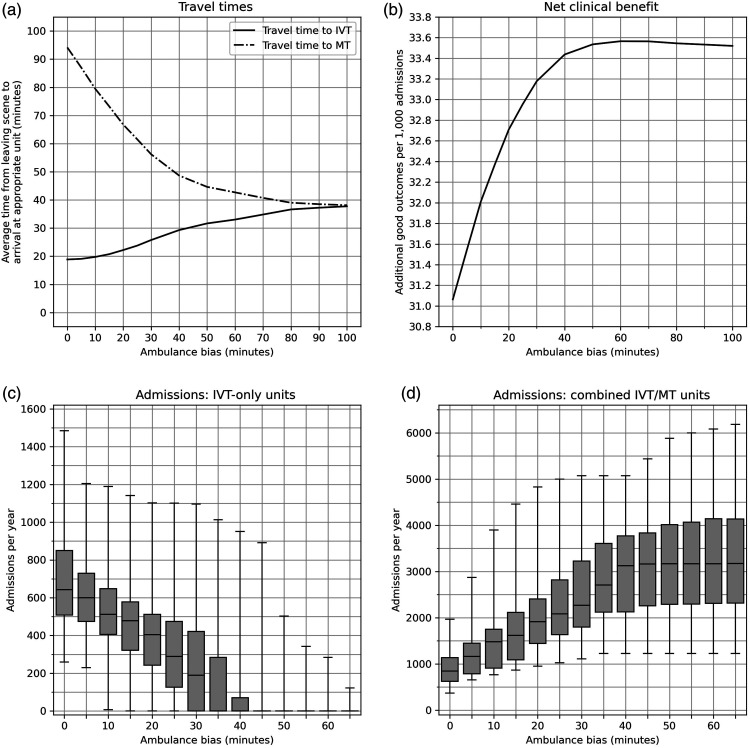
The effect of ambulance bias on travel time to IVT and MT (top left panel), clinical outcome (top right panel), first admission numbers to IVT-only units (bottom left panel) and first admission numbers to MT centres (bottom left panel). Ambulance bias is measured as the additional travel time an ambulance crew will allow to go directly to a MT centre instead of a closer IVT-only unit. Boxplots show median (mid-way line), interquartile range (box) and range (whiskers) for individual units. The model has 24 MT centres at the current neuroscience centres and 83 IVT-only units at the remaining current acute stroke centres.

So far we have examined the effect of bypassing a nearer IVT-only centre in order to directly attend an IVT/MT centre for all patients (mothership model), and just for those for whom it is expected that bypass will confer a better clinical outcome. Alternatively, we can assess the impact of allowing a maximum additional travel time in order for a patient to travel directly to an IVT/MT centre. In previous studies, we have introduced the concept of ambulance bias, defined as the additional time that an ambulance crew is willing to travel in order to convey a patient directly to an IVT/MT centre. As ambulance bias increases, a number of effects are observed (Figure 4): average time to IVT is increased, average time to MT is reduced, net clinical outcome is improved, admissions to IVT-only units fall and admissions to IVT/MT centres increase. For example, if ambulance crews are willing to travel 15 min further in order to directly attend an IVT/MT centre, the net benefit of reperfusion treatment increases from 31.1 to 32.3 additional good outcomes per 1000 admissions. However, the proportion of all stroke patients admitted directly to an IVT/MT centre would increase from 27% to 52%. The number of units with fewer than 300 first admissions per year increases from three to 18, while the number of units with fewer than 600 first admissions per year increases from 39 to 64 (out of 83 IVT-only units in total). At the same time, the number of units with more than 2000 first admissions increase from zero to eight.

This effect on admissions may again be mitigated by selecting those patients with a known stroke onset time, with arrival at hospital expected within 6 h from onset, and with a NIHSS > 5. With 15 min allowable extra time to reach the MT-capable centre the proportion of all stroke patients admitted directly to an IVT/MT centre would be 33%. Average first admissions to an IVT/MT centre would be 1127 (a 23% increase compared with all patients attending closest unit first), with the largest centre having 2574 admissions. Average first admissions to IVT-only units would be 649 (a 9% reduction compared with all patients attending closest unit first), with 4 IVT-only units receiving fewer than 300 admissions per year.

### Pre-hospital selection for large artery occlusion

Applying the RACE pre-hospital clinical diagnostic test for LAO, with all ‘RACE-positive’ patients taken directly to an IVT/MT centre, increases the number of additional disability-free outcomes from 31.1 to 33.4 per 1000 admissions. However, the proportion of all stroke patients admitted directly to an IVT/MT centre would increase from 27% to 62%. The number of units with fewer than 300 first admissions per year increases from three to 30, while the number of units with fewer than 600 first admissions per year increases from 39 to 74 (out of 83 IVT-only units in total). At the same time, the number of units with more than 2000 admissions increases from zero to eleven.

The effect on admissions may be mitigated further by selecting those patients with a known stroke onset time, with arrival at hospital expected within 6 h from onset. Assuming the proportion of patients with known stroke onset time, and their expected arrival time at hospital, are similar to patients with NIHSS of greater than 5, the proportion of all stroke patients admitted directly to an IVT/MT centre would be 47%. Average first admissions to an IVT/MT centre would be 1571 (a 71% increase compared with all patients attending closest unit first), with the largest centre having 2791 admissions. Average first admissions to IVT-only units would be 521 (a 27% reduction compared with all patients attending closest unit first), with 8 IVT-only units receiving fewer than 300 admissions per year.

### Increasing the number of thrombectomy units

**Figure 5.**
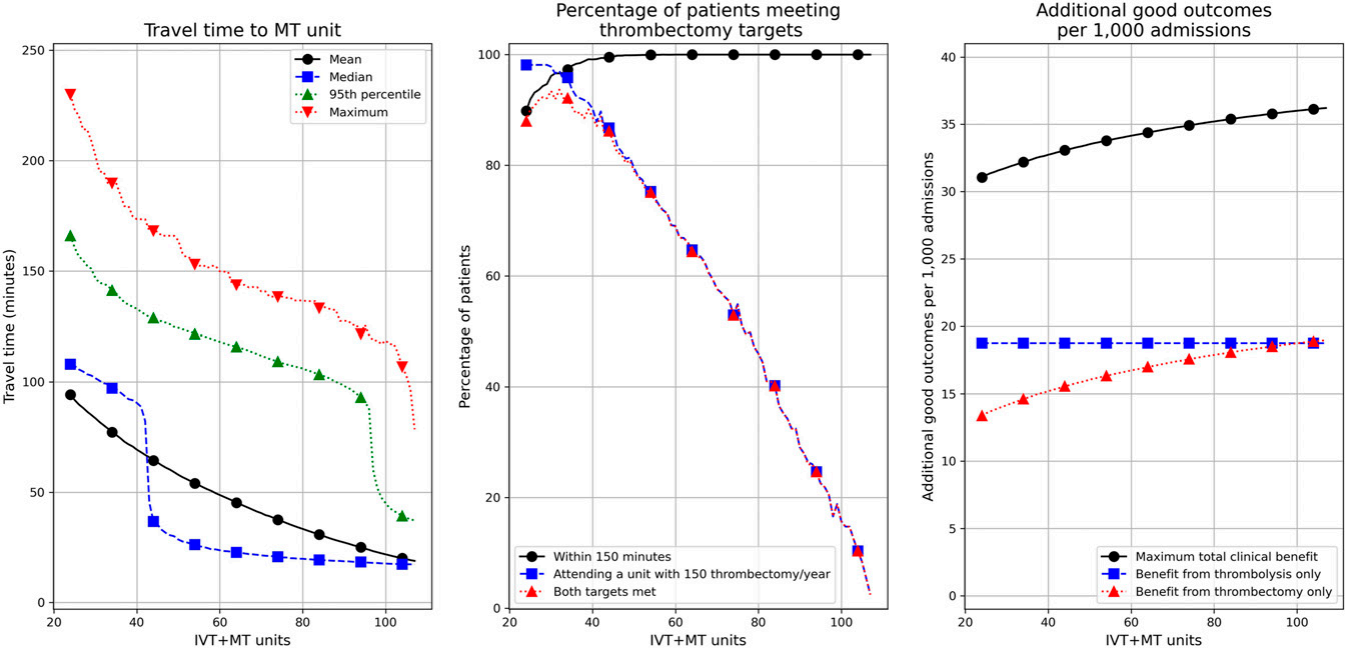
The effect of converting IVT-only units to MT centres. The charts show effect on travel time to a MT centre (left panel), the percentage of patients meeting MT admission/activity targets (middle panel), and the clinical benefit from reperfusion (right panel). The base case model has 24 MT centres at the current neuroscience centres and 83 IVT-only units at the remaining current acute stroke centres.

Our genetic algorithm identified the optimal locations of additional IVT/MT centres, in order to maximise access to MT while also limiting maximum admission numbers to all units (Figure 5). As the number of units providing both IVT and MT increases, time to MT reduces and the net clinical benefit of reperfusion treatment increases from 31.1 to 36.2 additional good outcomes per 1000 admissions with all 107 units providing both IVT and MT. However, beyond about 30 IVT/MT centres, the capacity to maintain 150 procedures per year at each centre markedly reduces. If all IVT/MT centres are required to perform at least 150 MT procedures per year the maximum clinical benefit, achieved with 29 IVT/MT centres, is 31.5 additional disability-free outcomes per 1000 admissions.

## Discussion

### Summary of findings and comparison to previous modelling

There have been a number of modelling studies on the relative benefits of mothership versus drip-and-ship.^17,27–30^ Our work applies previously described outcome-based modelling methods^17^ to a population of 52.5 million in England. Previous studies on outcome focus on using modelling to decide where best to take patients—to a closer IVT-only unit or a more distant MT-capable centre, though Saraj et al.^31^ also compared a bypass strategy with converting up to 20% of existing IVT-only units in the US to MT-capable units, and found that a 15 min bypass strategy (allowing an extra 15 min journey time to directly access MT) increased access to MT in the US more than converting 20% of existing IVT-only units to also provide MT. Our work uses modelling to examine outcomes, but also to examine the expected impact on acute stroke units with differing organisational strategies. While an individual patient-centred analysis of outcomes may favour one particular model, consideration must also be given to unintended potential destabilising effects of the strategies. Studies have looked at ways of managing the demand at MT centres,^32,33^ but less consideration has been given to the potential loss of experience and expertise in administering alteplase in IVT-only centres. Overwhelming MT-capable centres or depleting IVT-only centres of experience may both destabilise emergency stroke services leading ultimately to a sub-optimal solution for the patient population overall.

Our modelling is based on England, but the results are similar to those described in other countries and contexts. For example, Schlemm et al.^30^ found that patient outcomes across Germany would best be served by a mothership model of care across the country, and studies by Holodinsky et al.^34,35^ have shown that retaining IVT-only site adds value only if transport times to MT-capable centres are long and if the IVT-only centre can reach ‘ideal’ door-to-treatment times of 30 min or less. Minimising door-in-door-out times at IVT-only centres is essential to good performance of a drip-and-ship model of care.^36^ Similar to those studies, we have found that the population benefit from reperfusion is maximised when the proportion of units that are MT-capable is highest. Put another way, the closer the ultimate national configuration of acute stroke units is to a full mothership model, the greater the benefits achievable from reperfusion as a whole. This can be achieved either by reducing the number of IVT-only units, or by increasing the number of IVT/MT centres. There are however barriers to increasing the number of IVT/MT centres (such as capital cost, workforce and the time taken to establish infrastructure). While maximum patient benefit may be achieved by sending most people to the existing/planned 24 MT-capable centres, without effective pre-hospital selection this would produce admission numbers that are unsustainable. Local IVT-only centres are therefore required to manage demand at MT-capable centres and create a sustainable emergency stroke system.

### Ambulance bias or ‘bypass’

Although we have identified that a hybrid model of IVT-only units and IVT/MT centres is preferable to create a sustainable system, we have also identified some important factors that would destabilise any hybrid system. Principal among these is the natural inclination for ambulance paramedics to travel a little further in order to improve the chances of reperfusion treatment for the individual patient under their care, which we have called ‘ambulance bias’. This phenomenon is well recognised anecdotally, with some IVT/MT centres reporting ambulances travelling up to 30 min further to deliver their patient to a MT-capable site (C Roffe, personal communication). This strategy, also known as *‘bypass’,* has been recommended in US and European guidelines for suspected LAO.^37–39^ This has the net effect of converting any hybrid system towards a *de facto* mothership model, with a pronounced increase in the proportion of patients conveyed directly to an IVT/MT centre, and a corresponding reduction in admissions to IVT-only units, effectively negating the mitigating effect of having IVT-only units. These considerations of institutional viability (at either end of the scale for stroke admissions) have not featured in the reasoning behind other analyses based on additional transport time and bypass.^40^

### Pre-hospital patient selection to mitigate the destabilising effects of bypass

In our study we have first modelled a system where all patients will follow the drip-and-ship or mothership paradigm before examining methods of mitigating the significant shifts in admission numbers. In reality, many patients unsuitable for IVT or MT will not need transfer to a MT-capable centre. In England, 25% of patients with acute stroke have a NIHSS score of six or more and a known stroke onset time of less than 6 h before arrival at hospital,^6^ and so it may be considered that this subset of patients is the great majority of those eligible for direct transfer to a MT-capable centre. However, the proportion of patients who may be considered for MT has been expanded by the use of advanced imaging to include those with unknown onset time,^41^ onset-to-arrival times of up to 24 h,^42^ and milder strokes with a large radiographic perfusion deficit who are at higher risk of deterioration.^43^ If only those patients with a known stroke onset time more than 12 h ago are regarded as unsuitable for bypass, this would constitute just 13% of stroke patients.^6^ The proportion of patients that may be ruled out for direct transfer to a MT-capable centre is therefore likely to lie somewhere between 13% and 75% depending on the criteria and pre-hospital clinical diagnostic tests applied.

The pre-hospital selection of patients for bypass using clinical diagnostic tests for LAO is aimed at identifying those patients most suitable for MT. From among many such tests, we have used the RACE^21^ to exemplify pre-hospital selection because there is a good level of published detail available and it has also recently been shown to be the best performing among a range of 8 similar pre-hospital clinical scales,^44^ but other triage scales have been implemented, such as LAMS.^45^ Though RACE improves selection of LAO patients, we found applying RACE still has the potential to destabilise the current organisation of care through its low specificity; furthermore the latest trial data suggest that the widespread use of such pre-hospital selection does not confer the anticipated benefits in terms of improved outcomes from bypass and earlier treatment with MT.^40^ More sophisticated pre-hospital pathways, such as supporting paramedics with centralised triage^33^ or advances in technology, such as use of biomarkers^46^ or ultrasound devices^47^ for detecting LAO, may be necessary to achieve the requisite balance of sensitivity and specificity. Selection of patients eligible for MT will always selectively divert patients who are more likely to also receive IVT. For example, while 25% of patients may be selected for direct transfer to a MT-capable centre using the three criteria of a known stroke onset time, arrival at hospital within 6 h of known stroke onset and a NIHSS of >5, these patients account for 73% of thrombolysis use.^6^

### Alternative strategies

Other strategies for the widespread delivery of endovascular stroke therapy have been proposed, including mobile interventionist teams^48,49^ who travel to the patient’s nearest admitting hospital. We have not modelled these and restricted our study to those solutions that are under consideration across England and are likely to be implementable at a national scale within existing NHS infrastructure. In particular, the infrastructure costs of developing a much larger number of sites capable of intracranial endovascular interventions are prohibitive. For the UK, with its relatively low proportion of MT-capable sites (22% of all acutely admitting sites), the choice essentially lies between drip-and-ship or mothership models of care, or a combination of the two. Similarly, we have assumed patients are transferred by road, rather than by other means such as helicopter, although this may offer a feasible and cost-effective option for a few more remote populations if it reduces travel time by more than 60 min.^50^

### Model assumptions

We have based our model on assumptions regarding a reasonably achievable level of both IVT and MT, at 20% and 10% of all admitted stroke cases respectively.^8^ For IVT, these rates of treatment are observed in many regions and countries elsewhere in Europe^51,52^ but significantly more progress is needed in the UK to achieve this target as IVT treatment has remained unchanged at 11–12% over the last 7 years, with persisting five-fold variation between hospitals in use of thrombolysis.^2^ Our modelling assumes the MT is well established in the designated MT centres, and so provides a ‘final steady state’ view of the potential clinical benefit and admission numbers to different unit types. Currently across the UK MT is known to be established at 24 of 28 planned MT-capable centres^12^ though still only a minority of sites are presently operating 24/7. It may therefore be some time yet before the greater levels of population benefit identified in our study are realised in practice.

When modelling the effect of drip-and-ship or mother ship models, we assume that the only difference between these models is time to treatment; we assume that all units behave similarly and that there is no deleterious effect of transport other than it’s effect on time to treatment. These simplifications allow us to isolate those effects that are due to changes in times to treatment for IVT and MT. Local planning may need to take into account other factors, particularly thrombolysis use and speed at local hospitals. Studies of inter-hospital transfer for MT have concluded the general safety of transfer, though the most severe stroke patients being transferred large distances may benefit from the presence of a physician.^53,54^ The potential adverse impacts from large numbers of patients ineligible for reperfusion being displaced to a more remote site are poorly understood, but cannot be disregarded. Any such negative impacts have not been modelled in our study.

In order to isolate the effects of alternative strategies we have generally assumed all hospitals will perform equally well, but in our model we found a doubling of clinical benefit if thrombolysis rates across England were increased from the current rates to a target of 20%. Though this study focusses on modelling alternative strategies for where emergency stroke patients should go, it should not be forgotten that identifying the causes of poor performance and delays at individual hospitals remains important.^55,56^

### Study limitations

We have not directly modelled stroke mimics. Mimics may be identified at different stages of the pathway, by paramedics, in the emergency department, or after admission to a stroke unit. Published data for this latter category would suggest a rule of up to ‘one third again’ for mimics.^57,58^ While mimics should have little or no effect on the number of IVT and MT procedures, they will affect total ambulance conveyance numbers, and caseloads for emergency departments and stroke teams. Any changes to hospital destination with different emergency stroke strategies are likely to be amplified by the presence of stroke mimics.

We have modelled and presented clinical benefit purely in terms of disability-free survival, rather than reductions in other disability states. For instance, converting a patient from severe disability (mRS 4–5) to mild disability (mRS 2) is a significant gain and even reduction from severe to moderate disability (mRS 3) is advantageous to individuals and society. We have defined mRS 0–1 rather than 0–2 as a good outcome, whereas mRS 0–1 is in reality an excellent outcome; our modelling therefore presents a conservative picture of the population benefit from the widespread application of reperfusion treatments.

Our estimates of the time-dependent benefits of MT are based on the largest completed study of the effectiveness of MT in a national acute stroke system, the Dutch MR CLEAN randomised controlled trial.^59^ We used these data, in preference to the HERMES individual patient meta-analysis,^60^ on the understanding that MR CLEAN most closely represents the implementation of MT within the English NHS. The HERMES meta-analysis included several studies which used imaging-based selection of later-presenting patients, which has the effect of flattening the curve representing the decay of absolute benefit from MT over time.^61^ Currently this category of late-presenting patients represents very few patients treated in England, but as clinical practice develops with patients who are late-presenting or who have unknown onset time, this is likely to involve more MT-eligible, IVT-ineligible patients, and thus strengthen the case for a predominantly mothership model.

We have modelled only England in our study. While many of the principles (e.g. the advantages of direct transfer to MT except when travel times to MT are very long, and the potential destabilising effect of bypass for some patients away from their local IVT-only centre) will be applicable in many countries, the results presented pertain particularly to England. Separate modelling for other countries should be undertaken when planning services.

## Conclusions

Our study has used outcome-based modelling to compare the two principal service configurations for the wider availability of MT in the UK – drip-and-ship or mothership – and it has identified large potential reductions in population disability from greater access to reperfusion therapy. However, delivering these gains within a constrained system demands a balance between diverting excessive patient numbers to MT-capable sites and depleting IVT-only sites of activity and expertise. Current methods of patient selection including pre-hospital clinical diagnostic tests and bypass policies may destabilise such systems and at present do not offer methods that are sufficiently reliable to justify widespread adoption. Further research is needed to identify the best combination of pre-hospital selection and divert policies that confers the greatest population benefit within a sustainable acute care system.
